# ClinVAP: a reporting strategy from variants to therapeutic options

**DOI:** 10.1093/bioinformatics/btz924

**Published:** 2019-12-12

**Authors:** Bilge Sürün, Charlotta P I Schärfe, Mathew R Divine, Julian Heinrich, Nora C Toussaint, Lukas Zimmermann, Janina Beha, Oliver Kohlbacher

**Affiliations:** 1 Department of Computer Science, Applied Bioinformatics, Tübingen 72076, Germany; 2 NEXUS Personalized Health Technologies, ETH Zurich, Zurich 8093, Switzerland; 3 Translational Bioinformatics, University Hospital Tübingen, Tübingen 72076, Germany; 4 Center for Personalized Medicine, University Hospital Tübingen, Tübingen 72076, Germany

## Abstract

**Motivation:**

Next-generation sequencing has become routine in oncology and opens up new avenues of therapies, particularly in personalized oncology setting. An increasing number of cases also implies a need for a more robust, automated and reproducible processing of long lists of variants for cancer diagnosis and therapy. While solutions for the large-scale analysis of somatic variants have been implemented, existing solutions often have issues with reproducibility, scalability and interoperability.

**Results:**

Clinical Variant Annotation Pipeline (ClinVAP) is an automated pipeline which annotates, filters and prioritizes somatic single nucleotide variants provided in variant call format. It augments the variant information with documented or predicted clinical effect. These annotated variants are prioritized based on driver gene status and druggability. ClinVAP is available as a fully containerized, self-contained pipeline maximizing reproducibility and scalability allowing the analysis of larger scale data. The resulting JSON-based report is suited for automated downstream processing, but ClinVAP can also automatically render the information into a user-defined template to yield a human-readable report.

**Availability and implementation:**

ClinVAP is available at https://github.com/PersonalizedOncology/ClinVAP.

**Supplementary information:**

Supplementary data are available at *Bioinformatics* online.

## 1 Introduction

Understanding the genetic profile of a patient’s tumor to assess clinical actionability is a key to establish personalized targeted therapies. Large volumes of genomic data from cancer patients have become available due to the ever-decreasing costs of sequencing. Strategies need to be developed to gain insights from the data that can be used to support treatment decisions for individual patients. Identification of somatic variants that render a tumor either susceptible or resistant to treatment as well as detection of the genes driving a specific cancer can be essential for therapeutic decision making. Although there are publicly available databases to annotate genetic variants with respect to their actionability, it is time-consuming to query these resources manually. Furthermore, sending patient-related information to web services for therapeutic variant annotation undermines privacy preservation which hinders the use of services such as PharmGKB (Whirl-Carrillo *et al.*, 2012). On the other hand, local instances of clinical annotation (Wendl *et al.*, 2011) and reporting (Perera-Bel *et al.*, 2018) pipelines can be difficult to use. Common problems are complex command line interfaces, the necessity to modify the source code, the requirement of non-standard variant file formats, or the lack of structured output.

Here, we introduce our Clinical Variant Annotation Pipeline (ClinVAP) which annotates somatic single nucleotide variants given as standard VCF file with their driver gene type and relevant drug information. This information is prioritized based on actionability and severity of gene disruption. To this end, we supply the user with affected driver genes and direct as well as indirect drug targets by cross-referencing the observed variants with evidence from several public repositories. The resulting report provided as JSON, Microsoft Word, or PDF file can be helpful to discussion otherwise overlooked therapeutic options.

## 2 Materials and methods

### 2.1 Data integration

A clinical annotation knowledge base implemented as MongoDB database forms the annotation source of the pipeline. It was built based on the full set of genes contained in the HGNC and UniProt databases. Information on drug targets as well as genes initiating tumorigenesis was collated within the database. The knowledge base is queried by the reporting application for each mutated gene found by Ensembl Variant Effect Predictor (VEP).

**Annotation of driver genes.** A catalog of 1998 driver genes was assembled from the databases and from the literature (Supplementary Material SA, Section 2.1). A confidence score was calculated for each driver gene by counting the number of considered sources that include the corresponding gene, in order to present a simple assessment of its significance as a driver in the literature.

**Mechanistic drug-target relations.** Genes were further annotated with drug target data compiled from databases, and from manually curated dataset of molecular drug targets (Supplementary Material SA, Section 2.1). Analogously to the cancer driver data, a confidence score was generated that represents the number of sources supporting the drug-gene association.

### 2.2 Reporting application

We devised a fully automated and containerized pipeline which takes a VCF file version 4.0+ as input that by default should contain the somatic variants of a tumor sample and creates a clinical report. The multi-step process builds on Ensembl VEP for variant annotation, queries the knowledge base and processes the resulting annotated file in an R application to render the results into a machine- and/or human-readable report.

**Variant Effect Prediction.** The first step of the pipeline is to annotate the variants using Ensembl VEP v93 in offline mode (McLaren *et al.*, 2016). The annotations are conducted based on user provided genome assembly version, i.e. GRCh37, GRCh38. Functional effects of variants on the canonical transcripts are predicted using SIFT and Polyphen (Adzhubei *et al.*, 2010; Kumar *et al.*, 2009).

**Variant Annotation.** In this step, the descriptive and interpretive information on variants such as genomic position, variant effect, are retrieved from the VEP-annotated VCF file by the R-based reporting application. Among the different annotation blocks of the alternatively spliced variants, the ones that are selected per variant by VEP are used in the next steps. Variants that did not pass quality control in the variant calling pipeline used to produce the input VCF as well as variants that were predicted by VEP as non-coding or low-effect were excluded. Furthermore, the variants predicted as ‘tolerated’ or ‘tolerated low confidence’ by SIFT and ‘benign’ by PolyPhen were removed. Using HGNC identifiers of the remaining variants, the knowledge base is queried to provide information about driver genes and affected drug targets. Clinical evidence summaries from the CIViC database is further incorporated to report the variants with a direct impact on actionability (Griffith *et al.*, 2017). CIViC’s scoring schema is adopted in the application to provide a quick overview over the confidence of the provided association.

**Report Generation.** The variants that (i) occur in a known cancer driver gene, (ii) have been observed previously in the context of altered treatment response, or (iii) fall in the coding region of the mechanistic target gene of a cancer therapeutic, are distributed into four categories and saved as a JSON file (see tables in Supplementary Material SC). If desired, the JSON report can be rendered into a user-provided template (in Microsoft DOCX format) to obtain a human-readable document (Supplementary Material SB).

### 2.3 Deployment and benchmarking

ClinVAP is available as self-contained Docker and Singularity images (Supplementary Material SA, Section 3) (Kurtzer *et al.*, 2017; Merkel, 2014). Containerized execution of the pipeline ensures easier versioning, full reproducibility of results and convenient execution on large-scale datasets. In order to test the robustness and performance of ClinVAP, we processed 500 VCF files from 430 donors including simple somatic mutations from ICGC cancer projects (ICGC, 2010). Average runtime was on approximately 7 min per file and current hardware. The median number of driver genes per report was five, with individual donors having up to 200 driver genes. We identified therapeutic suggestions for 65.2% of the cases, where the CIViC evidence level is restricted to either A, B, or C. In an additional 28.8% of cases, predicted effects of variants were annotated, but no conclusive therapeutic option was identified. Only in 6% of the cases ClinVAP could not provide any helpful information at all.

## 3 Conclusion

We introduce a fully automated, fast and robust annotation pipeline designed to equip Molecular Tumor Boards with evidence-based patient reports. ClinVAP reports reveal the molecular driving forces in cancer formation along with actionable therapeutic targets from the respective tumor’s set of somatic variants. The container technologies Docker and Singularity allow for easy deployment and reproducibility. The pipeline is run locally and does not require any patient data to be analyzed by external web sites. Therefore, use of ClinVAP is conform with standard privacy and data security regulations.

## Supplementary Material

btz924_Supplementary_Data
