# Supplementary material for: ClinVAP: a reporting strategy from variants to therapeutic options
Source: Bioinformatics. 2019 Dec 12;36(7):2316–7. doi: 10.1093/bioinformatics/btz924 (PMC7141851; doi:10.1093/bioinformatics/btz924)
Supplement: btz924_Supplementary_Data [file btz924_supplementary_data.zip › btz924-suppl_data/SupplementaryMaterialA_PipelineDocumentation.pdf]

Clinical Variant Annotation Pipeline

October 24, 2019

Contents

1 Introduction **1**

2 Pipeline Components **1**
2.1 Annotation Knowledge Base 2
2.2 Reporting Application 2

3 Implementation and Availability **3**
3.1 Running Pipeline with Singularity 3
3.2 Running Pipeline with Docker 5
3.2.1 Implementation For Mac and Ubuntu Users 5
3.2.2 Implementation With Docker Toolbox For Windows Users 6
3.2.3 Customising Report Template with Docker 7

4 Software Availability **7**

1 Introduction

The Clinical Variant Annotation Pipeline (ClinVAP) uses simple somatic mutations (SNVs) of a patient's tumor given in VCF files and creates structured clinical reports by annotating, prioritizing, and filtering the genomic variants. The report is designed to assist Molecular Tumor Boards (MTB) in making therapeutic decisions by providing them with information on the molecular mechanisms initiating carcinogenesis and on actionable genes.

Disclaimer The report created by ClinVAP is intended as a hypothesis generating framework and thus for research use only. It is not intended for diagnostic or clinical purposes. Information provided in the report does not replace a physician's medical judgment and usage is entirely at your own risk. The providers of this resource shall in no event be liable for any direct, indirect, incidental, consequential, or exemplary damages.

2 Pipeline Components

The pipeline consists of two major components: the annotation knowledge base and the reporting application. The database is queried by the reporting application to annotate driver genes and to identify drug targets among the mutated genes. The reporting application uses the Ensembl Variant Effect Predictior (VEP) to predict the effect of somatic variants, an R-based processing to process, filter, and prioritize the variants, and docxtemplater to render the results into the report template.

2.1 Annotation Knowledge Base

Genes identified as drivers are annotated with their action type, i.e. Tumor suppressor gene (TSG) or Oncogene. They are further annotated with information on their targeting drugs. This information is incorporated into a MongoDB database and queried by the reporting application for every somatic variant from the patient's tumor.

Identification of driver genes. Information on the driver genes are collected from public databases and literature. A list of the sources used by ClinVAP and the number of driver genes obtained from them are given in Table 1 [14, 3, 2, 10, 12].

Table 1: Driver Gene Sources and Distribution

Source	Type	Gene Number
COSMIC v81	TSG	93
	Oncogene	118
	Unknown	364
	TSG/Oncogene	39
TSGene2.0	TSG	1214
UniProtKB	TSG	174
	Oncogene	233
	TSG/Oncogene	6
Rubio-Perez et al.	TSG	209
	Oncogene	184
	Unknown	83
Vogelstein et al.	TSG	72
	Oncogene	54

Identification of mechanistic drug targets. Information on drugs and their targets is collected from DrugBank 5.0.7, Therapeutic Target Database (TTD), The International Union of Basic and Clinical Pharmacology 2017.5 (IUPHAR), and Santos et al. [13, 8, 5, 11].

2.2 Reporting Application

Input type. The standard input type of ClinVAP is somatic variants given as VCF file. Please note that, if user has raw sequencing data, FASTQ files should be processed with an NGS pipeline to extract somatic variants in VCF format. We recommend SAREK pipeline which processes FASTQ files and reports the somatic and germline variants separately [4].

Ensembl VEP. The first step of the pipeline is to annotate the variants given in VCF file using Ensembl VEP v93 [9]. Please note that the VCF output should include somatic variants only. Depending on NGS pipeline that originally produced the VCF file, there should be somatic status information in its information field descriptions. If you have a mixed VCF file containing somatic and germline variants, please filter your input file according to the somatic status annotation. The annotation is conducted according to the human genome assembly version (GRCh37 or GRCh38) specified by user. SIFT and Polyphen are employed in VEP to reveal the effect of variants provided in the input file on protein function [6][1]. SIFT is used to identify whether an amino acid substitution leads to a non-synonymous coding single nucleotide polymorphism (nsSNP), and to categorize the mutations as "deleterious" (damaging) or "tolerated" [7]. PolyPhen software tool is used to label the variants as "probably damaging", "possibly damaging" or "benign"[1].

R Based Processing. The VEP output is further processed and filtered by the R-based script to create the report. Variants with effect classified as "low" or "moderate" are filtered along with those predicted by SIFT and PolyPhen as "tolerated" or "low confidence tolerated" and "benign". For the

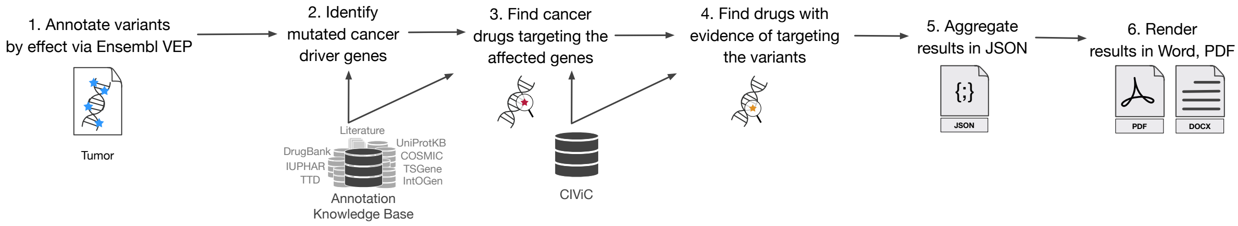

Figure 1: ClinVAP Workflow. It starts with variant effect prediction via Ensembl VEP tool which is followed by the identification of the driver genes and therapeutic targets of variants and affected genes by employing the annotation knowledge base. The results are outputted as *.JSON*, *.DOCX* and/or *.PDF* documents.

remaining variants, the database is queried to identify driver genes and cancer drugs targeting the affected genes. As an indication of the significance of the results, we calculated confidence score for both driver genes and the drug-gene pairs. The confidence score for driver genes shows the number of the background resources that listed the queried gene as a driver. The confidence score for the drug-gene pairs represents the number of references that contains the information on the association. Clinical evidence summaries from the CIViC database are also incorporated to find therapeutics that have evidence of targeting the genes and the observed variants. CIViC evidence levels (`https://civicdb.org/help/evidence/evidence-levels`) are incorporated to show the significance of the drug-variant-gene-disease associations. The results are outputted as *JSON* file. If available, patient metadata in *JSON* format is combined with the output file in the report generation step. If the diagnosis is provided within the metadata file, the pipeline returns gene-drug associations that is specific to the cancer type. Please use the disease dictionary file to find the correct disease ontology for the ClinVAP pipeline (`https://github.com/PersonalizedOncology/ClinVAP/blob/master/doc/disease_names_dictionary.txt`). Please note that for the application to identify the metadata, it has to have the same name as the input *VCF* file with *JSON* extension and to be structured as follows:

```
{
"patient_firstname":"<NAME>",
"patient_lastname":"<SURNAME>",
"patient_dateofbirth":"<DATE>",
"patient_diagnosis_short":"<DIAGNOSIS>",
"mutation_load":"<LOAD>"
}
```

Rendering report via docxtemplater tool. The JSON output of the R script is rendered into a word template(Figure 2).

3 Implementation and Availability

3.1 Running Pipeline with Singularity

On high performance computing clusters, the usage of Docker containers is usually not permitted due to security considerations. For this reason, ClinVAP is also offered as Singularity images.

Overview
Reports are generated via the reporting image that contains the database dump, VEP instance, and the reporting application. In order to run the VEP instance in offline mode, the data deployment

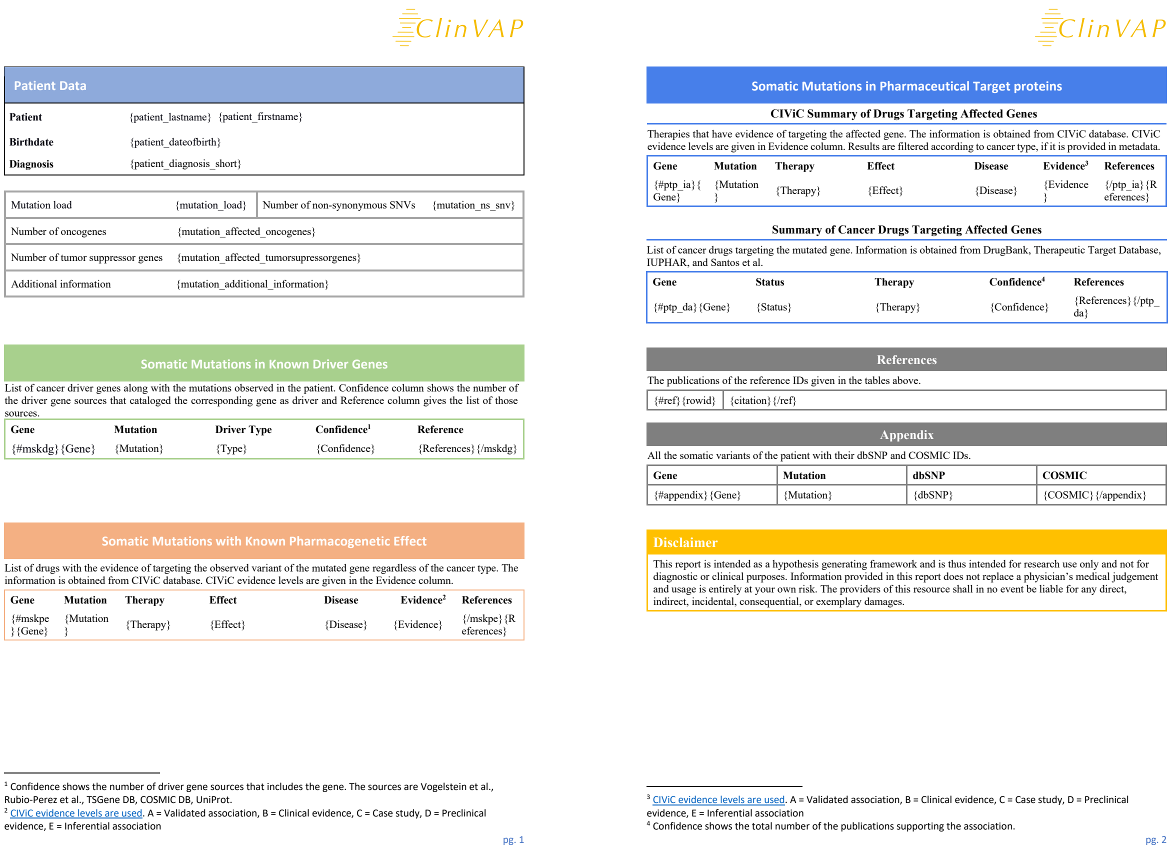

Figure 2: Report Template

image is used to transfer dependency files to the host volume.

System requirements

- Singularity 2.4+
- 12 GB of free space on home directory
- Availability of the ports 5000 and 27021, i.e. not being used by other application.

Availability

All images are publicly available on Singularity Hub at `https://www.singularity-hub.org/collections/2168`.

Implementation

1. Pull reporting image from Singularity Hub.

```
singularity pull -n reporting_app.img shub://
    PersonalizedOncology/ClinVAP:report
```

2. Pull dependency files image from Singularity Hub.

```
singularity pull -n file_deploy.img shub://PersonalizedOncology
   /ClinVAP:filedeploy
```

3. Run dependency files image first to transfer those file on your local folder.

```
singularity run -B /LOCAL/PATH/TO/FILES:/mnt file_deploy.img -a
    <Your Assembly Here>
```

4. Run the reporting image to generate the clinical reports.

```
singularity run -B /LOCAL/PATH/TO/FILES:/data -B /PATH/TO/INPUT
   /DATA:/inout reporting_app.img -t /inout -p jwp -a <Your
   Assembly Here>
```

3.2 Running Pipeline with Docker

Overview

The overall application consists of three Docker images for data deployment, the database, and report generation. The reporting image depends on the database and data deployment image. These three services are orchestrated via docker-compose with two shared volumes. One volume is used by the data deployment image to transfer the dependency files to the host system for enabling VEP to run offline. The second volume is used to provide input to the R application and to output the results to the same location.

System requirements

- Docker Engine release 1.13.0+
- Compose release 1.10.0+
- 34 GB of physical empty space on Docker Disk Image
- Availability of the ports 5000 and 27021, i.e. not being used by other application.

Availability

All images are publicly available on Docker Hub at `https://cloud.docker.com/u/personalizedoncology/repository/list`. The application orchestrates four images via docker-compose which are:

- Data deployment image, *clinvap_file_deploy*: Transfer the files that are necessary to run Ensembl VEP offline
- Database images, *clinvap_reporting_db* and *clinvap_reporting_db_api*: Starts the MongoDB database and Rest API service to conduct driver gene and mechanistic drug target annotation
- Reporting image, *clinvap_reporting_app*: Processes the input VCF file and outputs the report. Depends on the Data deployment image and the database image.

3.2.1 Implementation For Mac and Ubuntu Users

1. Clone Git repository

```
https://github.com/PersonalizedOncology/ClinVAP.git
```

2. Change into the cloned directory

```
cd ClinVAP/
```

3. Define assembly environmental variable for Docker

```
export ASSEMBLY=<Your Assembly Here>
```

4. Run the pipeline

```
docker-compose run -e ASSEMBLY --service-ports ClinicalReportR
   -t /inout -p jwp -a <Your Assembly Here>
```

$-a$: The genome assembly that was used in variant calling calling step to generate your VCF files. Valid values are:

$GRCh37$ for human genome assembly GRCh37

$GRCh38$ for human genome assembly GRCh38 $-t$: Directory hosting input files. It is handled by docker-compose file. Do not change this parameter.
$-p$: Output format to save the results. Select the corresponding argument here to get the report in specific format(s).

- j to save report in JSON format
- w to save report in DOCX format
- p to save report in PDF format

Resulting files should be in the host volume, *./ReportingApplication/inout* under the *ClinVAP* directory. Please note that volumes are handled by docker compose file.

3.2.2 Implementation With Docker Toolbox For Windows Users

Implementation only differs by the third command. Rest of the specifications are same.

1. Clone Git repository

```
https://github.com/PersonalizedOncology/ClinVAP.git
```

2. Change into the cloned directory

```
cd ClinVAP/
```

3. Define assembly environmental variable for Docker

```
export ASSEMBLY=<Your Assembly Here>
```

4. Run the pipeline

```
docker-compose run --service-ports ClinicalReportR -t //inout -
   p jwp -a <Your Assembly Here>
```

3.2.3 Customising Report Template with Docker

The report template is configurable according to the user preferences. It is possible to change the table orders, column orders within a table, change column names, remove tables or columns, include a logo etc. If you would like to change the mentioned settings, please follow the steps:

1. Clone Git repository, if you have not done so far.

   ```
   https://github.com/PersonalizedOncology/ClinVAP.git
   ```

2. Edit *template.docx* file under "ClinVAP/ReportingApplication/clinicalreporting_docxtemplater/data" and save the changes.

3. On terminal, change directory into "ClinVAP/ReportingApplication"

4. Build docker image to include the new template.

   ```
   docker build -t personalizedoncology/clinvap_reporting_app:v1.1 .
   ```

5. After successfully building the image, follow the implementation instructions for Docker.

4 Software Availability

The source code of ClinVAP is open source under MIT license and available on `https://github.com/PersonalizedOncology/ClinVAP`. If you would like to contribute, you may

- open an issue on GitHub,
- fork the repository, make changes and submit a pull request for us to review the changes and merge your contribution.

Please contact us on sueruen@informatik.uni-tuebingen.de for further information/help.

References

[1] Ivan A. Adzhubei et al. "A method and server for predicting damaging missense mutations". In: *Nature Methods* 7 (Apr. 2010). Correspondence, 248 EP -. URL: `http://dx.doi.org/10.1038/nmeth0410-248`.

[2] UniProt Consortium. "UniProt: the universal protein knowledgebase". In: *Nucleic acids research* 45.D1 (2016), pp. D158–D169.

[3] Simon A Forbes et al. "COSMIC: somatic cancer genetics at high-resolution". In: *Nucleic acids research* 45.D1 (2016), pp. D777–D783.

[4] Maxime Garcia et al. "Sarek: A portable workflow for whole-genome sequencing analysis of germline and somatic variants". In: *bioRxiv* (2018), p. 316976.

[5] Simon D Harding et al. "The IUPHAR/BPS Guide to PHARMACOLOGY in 2018: updates and expansion to encompass the new guide to IMMUNOPHARMACOLOGY". In: *Nucleic acids research* 46.D1 (2017), pp. D1091–D1106.

[6] Prateek Kumar, Steven Henikoff, and Pauline C. Ng. "Predicting the effects of coding non-synonymous variants on protein function using the SIFT algorithm". In: *Nature Protocols* 4 (June 2009), 1073 EP -. URL: `http://dx.doi.org/10.1038/nprot.2009.86`.

[7] Prateek Kumar, Steven Henikoff, and Pauline C Ng. "Predicting the effects of coding non-synonymous variants on protein function using the SIFT algorithm". In: *Nature protocols* 4.7 (2009), p. 1073.

[8] Ying Hong Li et al. “Therapeutic target database update 2018: enriched resource for facilitating bench-to-clinic research of targeted therapeutics”. In: *Nucleic acids research* 46.D1 (2017), pp. D1121–D1127.

[9] William McLaren et al. “The Ensembl Variant Effect Predictor”. In: *Genome Biology* 17.1 (June 2016), p. 122. ISSN: 1474-760X. DOI: 10.1186/s13059-016-0974-4. URL: https://doi.org/10.1186/s13059-016-0974-4.

[10] Carlota Rubio-Perez et al. “In silico prescription of anticancer drugs to cohorts of 28 tumor types reveals targeting opportunities”. In: *Cancer cell* 27.3 (2015), pp. 382–396.

[11] Rita Santos et al. “A comprehensive map of molecular drug targets”. In: *Nature reviews Drug discovery* 16.1 (2017), p. 19.

[12] Bert Vogelstein et al. “Cancer genome landscapes”. In: *science* 339.6127 (2013), pp. 1546–1558.

[13] David S Wishart et al. “DrugBank: a comprehensive resource for in silico drug discovery and exploration”. In: *Nucleic acids research* 34.suppl_1 (2006), pp. D668–D672.

[14] Min Zhao et al. “TSGene 2.0: an updated literature-based knowledgebase for tumor suppressor genes”. In: *Nucleic acids research* 44.D1 (2015), pp. D1023–D1031.
