# Supplementary material for: ClinVAP: a reporting strategy from variants to therapeutic options
Source: Bioinformatics. 2019 Dec 12;36(7):2316–7. doi: 10.1093/bioinformatics/btz924 (PMC7141851; doi:10.1093/bioinformatics/btz924)
Supplement: btz924_Supplementary_Data [file btz924_supplementary_data.zip › btz924-suppl_data/SupplementaryMaterialB_ExampleReport.pdf]

ClinVAP

## Patient Data

| | |
|---|---|
| **Patient** | DEF ABC |
| **Birthdate** | 12.12.1212 |
| **Diagnosis** | Melanoma |

| | | | |
|---|---|---|---|
| Mutation load | Medium | Number of non-synonymous SNVs | 131 |
| Number of oncogenes | 3 | | |
| Number of tumor suppressor genes | 6 | | |
| Additional information | | | |

## Somatic Mutations in Known Driver Genes

List of cancer driver genes along with the mutations observed in the patient. Confidence column shows the number of the driver gene sources that cataloged the corresponding gene as driver and Reference column gives the list of those sources.

| Gene | Mutation | Driver Type | Confidence[1] | Reference |
|---|---|---|---|---|
| BRAF | p.Val600Glu | Oncogene | 4 | 1,2,3,4 |
| SF3B1 | p.Pro718Leu | Oncogene | 3 | 1,2,4 |
| DLEC1 | p.Asp215Asn | TSG | 2 | 3,5 |
| FAM46C | p.Thr209Asn | unknown | 2 | 1,4 |
| GLI1 | p.Ser1094Phe | TSG/Oncogene | 2 | 3,5 |
| RPS6KA2 | p.Glu319Lys | TSG | 2 | 3,5 |
| ACHE | p.Thr95Ile | TSG | 1 | 5 |
| EPHB4 | p.Pro346Leu | TSG | 1 | 5 |
| ETV5 | p.Tyr445Cys | unknown | 1 | 1 |
| LPP | p.Ala119Gly | unknown | 1 | 1 |
| MADD | p.Ser1620Phe | TSG | 1 | 5 |
| PABPC3 | p.Gly234Arg | unknown | 1 | 4 |
| PCSK5 | p.Cys747Tyr | unknown | 1 | 4 |
| TNPO1 | p.Gln38His | unknown | 1 | 4 |

1 Confidence shows the number of driver gene sources that includes the gene. The sources are Vogelstein et al., Rubio-Perez et al., TSGene DB, COSMIC DB, UniProt.

# Somatic Mutations with Known Pharmacogenetic Effect

List of drugs with the evidence of targeting the observed variant of the mutated gene regardless of the cancer type. The information is obtained from CIViC database. CIViC evidence levels are given in the Evidence column.

| Gene | Mutation | Therapy | Effect | Disease | Evidence[2] | References |
|---|---|---|---|---|---|---|
| BRAF | V600E | Bevacizumab | Resistance | Colorectal Cancer | B | 31 |
| BRAF | V600E | Cetuximab | Resistance | Colorectal Cancer | B | 23 |
| BRAF | V600E | Cetuximab,Vemurafenib,Irinotecan | Sensitivity/Response | Colorectal Cancer | B | 30 |
| BRAF | V600E | Dabrafenib,Trametinib | Sensitivity/Response | Melanoma | B | 22 |
| BRAF | V600E | Vemurafenib | Sensitivity/Response | Melanoma | B | 25,29 |
| BRAF | V600E | Vemurafenib | Sensitivity/Response | Ovarian Cancer | B | 33 |
| BRAF | V600E | Vemurafenib,Cobimetinib | Sensitivity/Response | Melanoma | B | 27 |
| BRAF | V600E | Dabrafenib | Resistance | Non-small Cell Lung Carcinoma | C | 36 |
| BRAF | V600E | Dabrafenib,Trametinib DMSO | Sensitivity/Response | Cholangiocarcinoma | C | 52,53 |
| BRAF | V600E | Pertuzumab,Vemurafenib | Sensitivity/Response | Anaplastic Thyroid Carcinoma | C | 33 |
| BRAF | V600E | Pictilisib | Sensitivity/Response | Melanoma | C | 39 |
| BRAF | V600E | Trametinib DMSO,Dabrafenib | Sensitivity/Response | Cholangiocarcinoma | C | 54 |
| BRAF | V600E | Vemurafenib | Sensitivity/Response | Colorectal Cancer | C | 33 |
| BRAF | V600E | Vemurafenib | Sensitivity/Response | Ganglioglioma | C | 49 |
| BRAF | V600E | Vemurafenib | Sensitivity/Response | Laryngeal Squamous Cell Carcinoma | C | 33 |
| BRAF | V600E | Vemurafenib | Sensitivity/Response | Multiple Myeloma | C | 47,47 |
| BRAF | V600E | Vemurafenib | Sensitivity/Response | Ovarian Cystadenocarcinoma | C | 50 |
| BRAF | V600E | Vemurafenib | Sensitivity/Response | Papillary Thyroid Carcinoma | C | 42 |

2 CIViC evidence levels are used. A = Validated association, B = Clinical evidence, C = Case study, D = Preclinical evidence, E = Inferential association

# Somatic Mutations with Known Pharmacogenetic Effect

List of drugs with the evidence of targeting the observed variant of the mutated gene regardless of the cancer type. The information is obtained from CIViC database. CIViC evidence levels are given in the Evidence column.

| Gene | Mutation | Therapy | Effect | Disease | Evidence | References |
|---|---|---|---|---|---|---|
| BRAF | V600E | Vemurafenib,Panitumumab | Sensitivity/Response | Colorectal Cancer | C | 44 |
| BRAF | V600E | Vemurafenib,Panitumumab,Irinotecan | Sensitivity/Response | Cholangiocarcinoma | C | 55 |
| BRAF | V600E | BEZ235 (NVP-BEZ235, Dactolisib),GDC-0879 | Sensitivity/Response | Colorectal Cancer | D | 67 |
| BRAF | V600E | Capecitabine,Vemurafenib,Bevacizumab | Sensitivity/Response | Colorectal Cancer | D | 60 |
| BRAF | V600E | Cobimetinib | Sensitivity/Response | Cancer | D | 64 |
| BRAF | V600E | PLX4720,GDC0941 | Sensitivity/Response | Colorectal Cancer | D | 58 |
| BRAF | V600E | PLX4720,Nutlin-3 | Sensitivity/Response | Colorectal Cancer | D | 59 |
| BRAF | V600E | Selumetinib (AZD6244),BEZ235 (NVP-BEZ235, Dactolisib) | Sensitivity/Response | Melanoma | D | 63 |
| BRAF | V600E | Sorafenib,Panitumumab | Sensitivity/Response | Colorectal Cancer | D | 56 |
| BRAF | V600E | Vemurafenib | Resistance | Melanoma | D | 57 |
| BRAF | V600E | Vemurafenib | Sensitivity/Response | Colorectal Cancer | D | 60 |
| BRAF | V600E | Vemurafenib,Gefitinib,Cetuximab | Sensitivity/Response | Colorectal Cancer | D | 66 |

# Somatic Mutations in Pharmaceutical Target proteins

## CIViC Summary of Drugs Targeting Affected Genes

Therapies that have evidence of targeting the affected gene. The information is obtained from CIViC database. CIViC evidence levels are given in Evidence column. Results are filtered according to cancer type, if it is provided in metadata.

| Gene | Mutation | Therapy | Effect | Disease | Evidence[3] | References |
|---|---|---|---|---|---|---|
| BRAF | L505H | Vemurafenib | Resistance | Melanoma | B | 28 |
| BRAF | V600 | Dabrafenib | Sensitivity/Response | Melanoma | B | 26 |
| BRAF | V600 | RO4987655 | Sensitivity/Response | Melanoma | B | 24 |
| BRAF | V600D | Dabrafenib | Sensitivity/Response | Melanoma | B | 21 |
| BRAF | V600E | Dabrafenib,Trametinib | Sensitivity/Response | Melanoma | B | 22 |
| BRAF | V600E | Vemurafenib | Sensitivity/Response | Melanoma | B | 25,29 |
| BRAF | V600E | Vemurafenib,Cobimetinib | Sensitivity/Response | Melanoma | B | 27 |
| BRAF | V600K | Dabrafenib,Trametinib | Sensitivity/Response | Melanoma | B | 27 |
| BRAF | V600K | Vemurafenib | Sensitivity/Response | Melanoma | B | 25 |
| BRAF | AGK-BRAF | Sorafenib | Sensitivity/Response | Melanoma | C | 37 |
| BRAF | L597R | Vemurafenib | Sensitivity/Response | Melanoma | C | 38 |
| BRAF | V600 | BAY 86-9766 | Resistance | Melanoma | C | 40 |
| BRAF | V600E | Pictilisib | Sensitivity/Response | Melanoma | C | 39 |
| BRAF | V600E+V600M | Dabrafenib | Sensitivity/Response | Melanoma | C | 35 |
| BRAF | V600K | Vemurafenib | Sensitivity/Response | Melanoma | C | 41 |
| BRAF | AGK-BRAF | Vemurafenib | Resistance | Melanoma | D | 37 |
| BRAF | DEL 485-490 | LY3009120 | Sensitivity/Response | Cancer | D | 65 |
| BRAF | L505H | Vemurafenib | Resistance | Melanoma | D | 69 |
| BRAF | MUTATION | Trametinib | Sensitivity/Response | Cancer | D | 62 |
| BRAF | PAPSS1-BRAF | Trametinib | Sensitivity/Response | Melanoma | D | 61 |
| BRAF | PAPSS1-BRAF | Vemurafenib | Resistance | Melanoma | D | 61 |
| BRAF | TRIM24-BRAF | Trametinib | Sensitivity/Response | Melanoma | D | 61 |
| BRAF | V600D | Vemurafenib | Sensitivity/Response | Melanoma | D | 72,73 |
| BRAF | V600E | Cobimetinib | Sensitivity/Response | Cancer | D | 64 |
| BRAF | V600E | Selumetinib | Sensitivity/Response | Melanoma | D | 63 |

3 CIViC evidence levels are used. A = Validated association, B = Clinical evidence, C = Case study, D = Preclinical evidence, E = Inferential association

# Somatic Mutations in Pharmaceutical Target proteins

## CIViC Summary of Drugs Targeting Affected Genes

Therapies that have evidence of targeting the affected gene. The information is obtained from CIViC database. CIViC evidence levels are given in Evidence column. Results are filtered according to cancer type, if it is provided in metadata.

| Gene | Mutation | Therapy | Effect | Disease | Evidence | References |
|---|---|---|---|---|---|---|
| | | (AZD6244),BEZ235 (NVP-BEZ235, Dactolisib) | | | | |
| BRAF | V600E | Vemurafenib | Resistance | Melanoma | D | 57 |

## Summary of Cancer Drugs Targeting Affected Genes

List of cancer drugs targeting the mutated gene. Information is obtained from DrugBank, Therapeutic Target Database, IUPHAR, and Santos et al.

| Gene | Status | Therapy | Confidence[4] | References |
|---|---|---|---|---|
| BRAF | approved\|investigational | sorafenib | 9 | 6,7,8,9,10,11,12,13, 14 |
| BRAF | approved | dabrafenib | 4 | 12,14,15 |
| BRAF | approved | vemurafenib | 4 | 12,14,16,17 |
| BRAF | approved | regorafenib | 3 | 12,18 |
| TNFRSF8 | approved | brentuximab vedotin | 2 | 12,19 |
| BRAF | approved | gsk2118436 | 1 | 14 |
| BRAF | approved | r7204 | 1 | 14 |
| EPHB4 | approved | vandetanib | 1 | 12 |

## References

The publications of the reference IDs given in the tables above.


| | |
|---|---|
| 1 | Futreal et al., A census of human cancer genes., Nature reviews. Cancer, 4, 3, 2004 |
| 2 | Vogelstein et al., Cancer genome landscapes., Science (New York, N.Y.), 339, 6127, 2013 |
| 3 | Apweiler et al., UniProt: the Universal Protein knowledgebase., Nucleic acids research, 32, Database issue, 2004 |
| 4 | Rubio et al., In silico prescription of anticancer drugs to cohorts of 28 tumor types reveals targeting opportunities., Cancer cell, 27, 3, 2015 |
| 5 | Zhao et al., TSGene: a web resource for tumor suppressor genes., Nucleic acids research, 41, Database issue, 2013 |
| 6 | Flaherty et al., Chemotherapy and targeted therapy combinations in advanced melanoma., Clinical cancer research : an official journal of the American Association for Cancer Research, 12, 7 Pt 2, 2006 |
| 7 | Haluska et al., Therapeutic targets in melanoma: map kinase pathway., Current oncology reports, 8, 5, 2006 |
| 8 | Kim et al., Sorafenib inhibits the angiogenesis and growth of orthotopic anaplastic thyroid |


4 Confidence shows the total number of the publications supporting the association.

| References | |
|---|---|
| | carcinoma xenografts in nude mice., Molecular cancer therapeutics, 6, 6, 2007 |
| 9 | Eisen et al., Sorafenib in advanced melanoma: a Phase II randomised discontinuation trial analysis., British journal of cancer, 95, 5, 2006 |
| 10 | Lu et al., Sorafenib induces growth inhibition and apoptosis of human chondrosarcoma cells by blocking the RAF/ERK/MEK pathway., Journal of surgical oncology, 102, 7, 2010 |
| 11 | Chen et al., TTD: Therapeutic Target Database., Nucleic acids research, 30, 1, 2002 |
| 12 | Santos et al., A comprehensive map of molecular drug targets., Nature reviews. Drug discovery, 16, 1, 2017 |
| 13 | Wilhelm et al., BAY 43-9006 exhibits broad spectrum oral antitumor activity and targets the RAF/MEK/ERK pathway and receptor tyrosine kinases involved in tumor progression and angiogenesis., Cancer research, 64, 19, 2004 |
| 15 | Gibney et al., Clinical development of dabrafenib in BRAF mutant melanoma and other malignancies., Expert opinion on drug metabolism & toxicology, 9, 7, 2013 |
| 16 | Jordan et al., Vemurafenib  for the treatment of melanoma., Expert opinion on pharmacotherapy, 13, 17, 2012 |
| 17 | Wang et al., Conformation-specific effects of Raf kinase inhibitors., Journal of medicinal chemistry, 55, 17, 2012 |
| 18 | Zambon et al., Small molecule inhibitors of BRAF in clinical trials., Bioorganic & medicinal chemistry letters, 22, 2, 2012 |
| 19 | Francisco et al., cAC10-vcMMAE, an anti-CD30-monomethyl auristatin E conjugate with potent and selective antitumor activity., Blood, 102, 4, 2003 |
| 20 | Peeters et al., Massively parallel tumor multigene sequencing to evaluate response to panitumumab in a randomized phase III study of metastatic colorectal cancer., Clinical cancer research : an official journal of the American Association for Cancer Research, 19, 7, 2013 |
| 21 | Ponti et al., The somatic affairs of BRAF: tailored therapies for advanced malignant melanoma and orphan non-V600E (V600R-M) mutations., Journal of clinical pathology, 66, 5, 2013 |
| 22 | Menzies et al., Dabrafenib and trametinib, alone and in combination for BRAF-mutant metastatic melanoma., Clinical cancer research : an official journal of the American Association for Cancer Research, 20, 8, 2014 |
| 23 | De et al., Effects of KRAS, BRAF, NRAS, and PIK3CA mutations on the efficacy of cetuximab plus chemotherapy in chemotherapy-refractory metastatic colorectal cancer: a retrospective consortium analysis., The Lancet. Oncology, 11, 8, 2010 |
| 24 | Zimmer et al., Phase I expansion and pharmacodynamic study of the oral MEK inhibitor RO4987655 (CH4987655) in selected patients with advanced cancer with RAS-RAF mutations., Clinical cancer research : an official journal of the American Association for Cancer Research, 20, 16, 2014 |
| 25 | McArthur et al., Safety and efficacy of vemurafenib in BRAF(V600E) and BRAF(V600K) mutation-positive melanoma (BRIM-3): extended follow-up of a phase 3, randomised, open-label study., The Lancet. Oncology, 15, 3, 2014 |
| 26 | Falchook et al., Dabrafenib in patients with melanoma, untreated brain metastases, and other solid tumours: a phase 1 dose-escalation trial., Lancet (London, England), 379, 9829, 2012 |
| 27 | Larkin et al., Combined vemurafenib and cobimetinib in BRAF-mutated melanoma., The New England journal of medicine, 371, 20, 2014 |
| 28 | Hoogstraat et al., Detailed imaging and genetic analysis reveal a secondary BRAF(L505H) resistance mutation and extensive intrapatient heterogeneity in metastatic BRAF mutant melanoma patients treated with vemurafenib., Pigment cell & melanoma research, 28, 3, 2015 |
| 29 | Flaherty et al., Inhibition of mutated, activated BRAF in metastatic melanoma., The New England |

| References | |
|---|---|
| | journal of medicine, 363, 9, 2010 |
| 30 | Hong et al., Phase IB Study of Vemurafenib in Combination with Irinotecan and Cetuximab in Patients with Metastatic Colorectal Cancer with BRAFV600E Mutation., Cancer discovery, 6, 12, 2016 |
| 31 | Tol et al., BRAF mutation in metastatic colorectal cancer., The New England journal of medicine, 361, 1, 2009 |
| 32 | Hyman et al., Vemurafenib in Multiple Nonmelanoma Cancers with BRAF V600 Mutations., The New England journal of medicine, 373, 8, 2015 |
| 33 | Hainsworth et al., Targeted Therapy for Advanced Solid Tumors on the Basis of Molecular Profiles: Results From MyPathway, an Open-Label, Phase IIa Multiple Basket Study., Journal of clinical oncology : official journal of the American Society of Clinical Oncology, 36, 6, 2018 |
| 34 | Souglakos et al., Prognostic and predictive value of common mutations for treatment response and survival in patients with metastatic colorectal cancer., British journal of cancer, 101, 3, 2009 |
| 35 | Ponti et al., Overwhelming response to Dabrafenib in a patient with double BRAF mutation (V600E; V600M) metastatic malignant melanoma., Journal of hematology & oncology, 5, , 2012 |
| 36 | Rudin et al., Molecular characterization of acquired resistance to the BRAF inhibitor dabrafenib in a patient with BRAF-mutant non-small-cell lung cancer., Journal of thoracic oncology : official publication of the International Association for the Study of Lung Cancer, 8, 5, 2013 |
| 37 | Botton et al., Recurrent BRAF kinase fusions in melanocytic tumors offer an opportunity for targeted therapy., Pigment cell & melanoma research, 26, 6, 2013 |
| 38 | Bahadoran et al., Major clinical response to a BRAF inhibitor in a patient with a BRAF L597R-mutated melanoma., Journal of clinical oncology : official journal of the American Society of Clinical Oncology, 31, 19, 2013 |
| 39 | Sarker et al., First-in-human phase I study of pictilisib (GDC-0941), a potent pan-class I phosphatidylinositol-3-kinase (PI3K) inhibitor, in patients with advanced solid tumors., Clinical cancer research : an official journal of the American Association for Cancer Research, 21, 1, 2015 |
| 40 | Weekes et al., Multicenter phase I trial of the mitogen-activated protein kinase 1/2 inhibitor BAY 86-9766 in patients with advanced cancer., Clinical cancer research : an official journal of the American Association for Cancer Research, 19, 5, 2013 |
| 41 | Sahadudheen et al., Long Term Survival and Continued Complete Response of Vemurafenib in a Metastatic Melanoma Patient with BRAF V600K Mutation., Case reports in oncological medicine, 2016, , 2016 |
| 42 | Ali et al., Extended Antitumor Response of a BRAF V600E Papillary Thyroid Carcinoma to Vemurafenib., Case reports in oncology, 7, 2, 2014 |
| 43 | Menzies et al., Clinical activity of the MEK inhibitor trametinib in metastatic melanoma containing BRAF kinase fusion., Pigment cell & melanoma research, 28, 5, 2015 |
| 44 | Pietrantonio et al., MET-Driven Resistance to Dual EGFR and BRAF Blockade May Be Overcome by Switching from EGFR to MET Inhibition in BRAF-Mutated Colorectal Cancer., Cancer discovery, 6, 9, 2016 |
| 45 | Grisham et al., Extreme Outlier Analysis Identifies Occult Mitogen-Activated Protein Kinase Pathway Mutations in Patients With Low-Grade Serous Ovarian Cancer., Journal of clinical oncology : official journal of the American Society of Clinical Oncology, 33, 34, 2015 |
| 46 | Subbiah et al., Targeted therapy by combined inhibition of the RAF and mTOR kinases in malignant spindle cell neoplasm harboring the KIAA1549-BRAF fusion protein., Journal of hematology & oncology, 7, , 2014 |
| 47 | Sharman et al., Vemurafenib response in 2 patients with posttransplant refractory BRAF V600E-mutated multiple myeloma., Clinical lymphoma, myeloma & leukemia, 14, 5, 2014 |

| References | |
|---|---|
| 48 | Ahronian et al., Clinical Acquired Resistance to RAF Inhibitor Combinations in BRAF-Mutant Colorectal Cancer through MAPK Pathway Alterations., Cancer discovery, 5, 4, 2015 |
| 49 | del et al., Response of recurrent BRAFV600E mutated ganglioglioma to Vemurafenib as single agent., Journal of translational medicine, 12, , 2014 |
| 50 | Combe et al., Sustained response to vemurafenib in a low grade serous ovarian cancer with a BRAF V600E mutation., Investigational new drugs, 33, 6, 2015 |
| 51 | Hsu et al., Mutations of KRAS/NRAS/BRAF predict cetuximab resistance in metastatic colorectal cancer patients., Oncotarget, 7, 16, 2016 |
| 52 | Kocsis et al., Combined dabrafenib and trametinib treatment in a case of chemotherapy-refractory extrahepatic BRAF V600E mutant cholangiocarcinoma: dramatic clinical and radiological response with a confusing synchronic new liver lesion., Journal of gastrointestinal oncology, 8, 2, 2017 |
| 53 | Lavingia et al., Impressive response to dual <i>BRAF</i> and MEK inhibition in patients with BRAF mutant intrahepatic cholangiocarcinoma-2 case reports and a brief review., Journal of gastrointestinal oncology, 7, 6, 2016 |
| 54 | Loaiza et al., Dramatic response to dabrafenib and trametinib combination in a BRAF V600E-mutated cholangiocarcinoma: implementation of a molecular tumour board and next-generation sequencing for personalized medicine., Ecancermedicalscience, 8, , 2014 |
| 55 | Silkin et al., Complete Clinical Response of BRAF-Mutated Cholangiocarcinoma to Vemurafenib, Panitumumab, and Irinotecan., Journal of gastrointestinal cancer, 47, 4, 2016 |
| 56 | Di et al., Wild-type BRAF is required for response to panitumumab or cetuximab in metastatic colorectal cancer., Journal of clinical oncology : official journal of the American Society of Clinical Oncology, 26, 35, 2008 |
| 57 | Nissan et al., Loss of NF1 in cutaneous melanoma is associated with RAS activation and MEK dependence., Cancer research, 74, 8, 2014 |
| 58 | Rad et al., A genetic progression model of Braf(V600E)-induced intestinal tumorigenesis reveals targets for therapeutic intervention., Cancer cell, 24, 1, 2013 |
| 59 | Ji et al., Vemurafenib synergizes with nutlin-3 to deplete survivin and suppresses melanoma viability and tumor growth., Clinical cancer research : an official journal of the American Association for Cancer Research, 19, 16, 2013 |
| 60 | Yang et al., Antitumor activity of BRAF inhibitor vemurafenib in preclinical models of BRAF-mutant colorectal cancer., Cancer research, 72, 3, 2012 |
| 61 | Hutchinson et al., BRAF fusions define a distinct molecular subset of melanomas with potential sensitivity to MEK inhibition., Clinical cancer research : an official journal of the American Association for Cancer Research, 19, 24, 2013 |
| 62 | Jing et al., Comprehensive predictive biomarker analysis for MEK inhibitor GSK1120212., Molecular cancer therapeutics, 11, 3, 2012 |
| 63 | Penna et al., Primary cross-resistance to BRAFV600E-, MEK1/2- and PI3K/mTOR-specific inhibitors in BRAF-mutant melanoma cells counteracted by dual pathway blockade., Oncotarget, 7, 4, 2016 |
| 64 | Hatzivassiliou et al., Mechanism of MEK inhibition determines efficacy in mutant KRAS- versus BRAF-driven cancers., Nature, 501, 7466, 2013 |
| 65 | Chen et al., Oncogenic BRAF Deletions That Function as Homodimers and Are Sensitive to Inhibition by RAF Dimer Inhibitor LY3009120., Cancer discovery, 6, 3, 2016 |
| 66 | Prahallad et al., Unresponsiveness of colon cancer to BRAF(V600E) inhibition through feedback activation of EGFR., Nature, 483, 7387, 2012 |
| 67 | Coffee et al., Concomitant BRAF and PI3K/mTOR blockade is required for effective treatment of BRAF(V600E) colorectal cancer., Clinical cancer research : an official journal of the American Association for Cancer Research, 19, 10, 2013 |

## References


| | |
|---|---|
| 68 | Dahlman et al., BRAF(L597) mutations in melanoma are associated with sensitivity to MEK inhibitors., Cancer discovery, 2, 9, 2012 |
| 69 | Wagenaar et al., Resistance to vemurafenib resulting from a novel mutation in the BRAFV600E kinase domain., Pigment cell & melanoma research, 27, 1, 2014 |
| 70 | Bertotti et al., A molecularly annotated platform of patient-derived xenografts ("xenopatients") identifies HER2 as an effective therapeutic target in cetuximab-resistant colorectal cancer., Cancer discovery, 1, 6, 2011 |
| 71 | NA et al., NA, NA, NA, NA, NA |
| 72 | Tsai et al., Discovery of a selective inhibitor of oncogenic B-Raf kinase with potent antimelanoma activity., Proceedings of the National Academy of Sciences of the United States of America, 105, 8, 2008 |
| 73 | Yang et al., RG7204 (PLX4032), a selective BRAFV600E inhibitor, displays potent antitumor activity in preclinical melanoma models., Cancer research, 70, 13, 2010 |
| 74 | Smalley et al., CRAF inhibition induces apoptosis in melanoma cells with non-V600E BRAF mutations., Oncogene, 28, 1, 2009 |
| 75 | Corcoran et al., BRAF gene amplification can promote acquired resistance to MEK inhibitors in cancer cells harboring the BRAF V600E mutation., Science signaling, 3, 149, 2010 |
| 76 | Heidorn et al., Kinase-dead BRAF and oncogenic RAS cooperate to drive tumor progression through CRAF., Cell, 140, 2, 2010 |


## Appendix

All the somatic variants of the patient with their dbSNP and COSMIC IDs.

| Gene | Mutation | dbSNP | COSMIC |
|---|---|---|---|
| TNFRSF8 | p.Pro215Ser | rs267597959 | COSM14024 |
| FAM46C | p.Thr209Asn | | |
| S100A7A | p.Gly98Trp | rs267598049 | COSM36721 |
| PKLR | p.Gly251Ser | rs267598065 | COSM36782 |
| MAEL | p.Ser431Cys | rs267598149 | COSM36684 |
| ZBTB41 | p.Phe164Val | rs267598277 | COSM36692 |
| SYT14 | p.Ser437Phe | rs267598356 | COSM36786 |
| OR2T8 | p.Met197Arg | rs4474294 | |
| OR2T3 | p.Ala214Thr | rs1770109 | |
| SLC4A5 | p.Ser428Phe | rs111392973 | COSM2999241,COSM2999242 |
| SLC4A5 | p.Ser428Thr | rs267599454 | |
| SNRNP200 | p.Arg1538Cys | rs267599495 | COSM36589 |
| SEMA4C | p.Arg407Trp | rs267599501 | COSM36666 |
| ANKRD36 | p.Ser1120Cys | rs768768868 | |
| KIAA1211L | p.Gly746Glu | rs866719486 | |
| DPP10 | p.Ile93Asn | | |
| XIRP2 | p.Gly127Arg | rs267598980 | COSM36673 |

| Appendix | | | |
|---|---|---|---|
| TTN | p.Pro10904Ser | rs267599054 | |
| TTN | p.Ala1347Thr | rs267599092 | COSM2708938,COSM2708939,COSM2708940,COSM2708941,COSM2708942 |
| SF3B1 | p.Pro718Leu | rs267599150 | COSM36655 |
| FZD7 | p.Pro285Ser | rs267599158 | COSM24315 |
| ZDBF2 | p.Gly575Arg | | |
| AGFG1 | p.Gly364Arg | rs267599235 | COSM25632,COSM3364621,COSM3364622 |
| ARL4C | p.Gly71Ser | rs61752230 | COSM21657 |
| KIF1A | p.Ser141Ala | | |
| DLEC1 | p.Asp215Asn | rs149190717 | COSM1566798,COSM36702 |
| VPRBP | p.Pro309Leu | rs267599884 | |
| TLR9 | p.Gly514Ser | rs267599888 | COSM36649 |
| PRR23C | p.Glu262Lys | rs759730911 | COSM36858 |
| CLSTN2 | p.Gln262His | rs267599628 | COSM36631 |
| SAMD7 | p.Arg67Trp | rs191885635 | COSM36663 |
| GNB4 | p.Pro107Leu | rs267599699 | COSM13667 |
| ETV5 | p.Tyr445Cys | rs267599722 | COSM23333 |
| LPP | p.Ala119Gly | | |
| MUC4 | p.Pro1056His | rs753583962 | |
| EXOC1 | p.Pro774Ser | rs267600192 | COSM36662 |
| REST | p.Pro752Thr | rs267600197 | COSM24349 |
| SMR3B | p.Arg58Lys | rs267600235 | COSM1310225,COSM36745 |
| ADAM29 | p.Gly589Glu | rs267600094 | COSM26290 |
| CARD6 | p.Leu638Phe | rs267600630 | COSM14006 |
| TNPO1 | p.Gln38His | rs267600680 | COSM36775,COSM5648790 |
| F2RL2 | p.Leu141Phe | rs267600693 | COSM27249,COSM36780 |
| VCAN | p.Asp203Asn | rs267600718 | COSM36758 |
| EDIL3 | p.Gln187Lys | rs267600722 | COSM26295 |
| PCDHB7 | p.Asp374His | | |
| GRIA1 | p.Gly828Glu | rs267600500 | COSM36714,COSM4854071,COSM4854072 |
| ADAM19 | p.Pro900Leu | rs61757467 | |
| MBOAT1 | p.Lys293Asn | | |
| SPDEF | p.Asp283His | | |

| Appendix | | | |
|---|---|---|---|
| SPDEF | p.Ser229Leu | rs200344679 | COSM36760 |
| SCUBE3 | p.Gly702Glu | rs267600995 | COSM36691 |
| BTBD9 | p.Arg46Cys | rs267601008 | COSM36793 |
| GPR111 | p.Ile290Leu | rs267601055 | COSM36619 |
| BAI3 | p.Asp755Asn | rs267601102 | COSM22119 |
| KATNA1 | p.Pro241Leu | rs267600852 | |
| KATNA1 | p.Pro241Ser | rs267600853 | |
| RPS6KA2 | p.Glu319Lys | rs267600891 | COSM21036,COSM3024932,COSM3024933 |
| ANLN | p.Gln649Arg | rs267601502 | COSM36632 |
| ABCA13 | p.Gly4948Asp | rs267601533 | COSM36817 |
| EPHB4 | p.Pro346Leu | rs267601191 | COSM21032 |
| ACHE | p.Thr95Ile | rs267601193 | COSM36706 |
| BRAF | p.Val600Glu | rs113488022 | COSM18443,COSM476,COSM6137 |
| RP11-1220K2.2 | p.Asp1426Glu | | |
| TRBV23-1 | p.Pro27Leu | | COSM36861 |
| ZNF862 | p.Gln583Lys | rs267601404 | COSM36833 |
| NAT2 | p.Glu264Lys | rs267601842 | COSM36677 |
| SCARA5 | p.Glu270Lys | rs267601883 | COSM36713 |
| GPR124 | p.Glu863Lys | rs267601912 | COSM36641 |
| REXO1L1P | p.Ser639Phe | | |
| CNBD1 | p.Leu135Arg | | |
| GRHL2 | p.Ser356Phe | rs267601682 | COSM36601 |
| ZC3H3 | p.Ser879Phe | rs267601811 | COSM36642 |
| ANKRD18A | p.Glu654Lys | rs267602244 | COSM36859 |
| PCSK5 | p.Cys747Tyr | rs267602276 | COSM36640 |
| NUTM2G | p.Gly36Asp | rs267602327 | COSM36612 |
| OR1J1 | p.Leu157Phe | rs267602118 | COSM36710 |
| GAPVD1 | p.Leu35Phe | rs267602131 | COSM36617 |
| ADAMTS13 | p.Arg398His | rs121908471 | COSM36777 |
| LHX3 | p.Gly92Glu | | COSM36599 |
| MADD | p.Ser1620Phe | rs267602903 | COSM26934 |
| OR4S2 | p.Arg120Cys | rs267602971 | COSM36685 |
| OR4D11 | p.Pro58Ala | rs267603040 | COSM36624 |
| SPTBN2 | p.Glu2047Lys | rs201985455 | COSM36751,COSM4199893 |
| GRM5 | p.Glu941Lys | rs267603229 | |

| Appendix | | | |
|---|---|---|---|
| DCP1B | p.Pro98Ser | rs267603408 | COSM36575 |
| CD163 | p.Pro310Leu | rs267603681 | COSM36725 |
| GLI1 | p.Ser1094Phe | rs267603606 | COSM24658 |
| TBC1D30 | p.Gly327Glu | rs267603627 | COSM36841 |
| KCNC2 | p.Leu298Ser | rs267603669 | COSM36754 |
| PABPC3 | p.Gly234Arg | rs267603790 | COSM36646 |
| CPB2 | p.Phe409Ser | rs267603833 | COSM36708 |
| CARKD | p.Pro205Ser | rs267603758 | COSM36577 |
| NOVA1 | p.Ala256Asp | rs267603974 | COSM1369439,COSM25331 |
| ARHGAP5 | p.Thr437Ile | rs56259828 | |
| SERPINA6 | p.Arg282Leu | rs267604111 | COSM1265285,COSM26307,COSM267404 |
| NUDT14 | p.Thr44Pro | rs267603899 | COSM36696 |
| IGHV1-18 | p.Gln20Lys | | |
| TRPM1 | p.Glu1261Lys | rs267604151 | COSM36625 |
| PLIN1 | p.Leu191Arg | | COSM36595 |
| PRC1 | p.Gly507Glu | rs267604387 | COSM36743 |
| AMDHD2 | p.His587Arg | | |
| NLRC3 | p.Gly454Arg | rs267604538 | COSM36804 |
| TTLL6 | p.Arg280Lys | rs267604932 | COSM36852 |
| USH1G | p.Leu379Ser | rs267605044 | COSM36661 |
| PSMA8 | p.Gly36Glu | rs267605136 | COSM36586 |
| MBD3 | p.Asp283Asn | rs369581342 | |
| GTF2F1 | p.Gly411Arg | | |
| MUC16 | p.Met2821Ile | rs267605807 | COSM2701120,COSM36853 |
| MUC16 | p.Leu2819Met | | |
| MUC16 | p.Leu1434Ile | | |
| OLFM2 | p.Arg58Gln | rs267605828 | COSM36654 |
| PKN1 | p.Arg191Cys | rs267605306 | COSM21035 |
| CYP4F2 | p.Arg149Gln | rs140630977 | COSM1129961 |
| ZNF208 | p.His855Tyr | rs267605385 | |
| ARHGAP33 | p.Pro1068Leu | | |
| NOSIP | p.Pro297Leu | | |
| ZNF880 | p.Pro169Gln | rs267605631 | COSM1234752 |
| ZSCAN5A | p.Ala179Thr | | |
| FLRT3 | p.Ile532Asn | | |

| Appendix | | | |
|---|---|---|---|
| DLGAP4 | p.Ala879Ser | rs267605913 | COSM36648 |
| SPO11 | p.Gly88Ser | rs267606012 | COSM36690 |
| TMEM50B | p.Ser113Phe | rs267606110 | COSM36665 |
| CECR2 | p.Gly474Arg | rs267606173 | COSM36851 |
| IGLV3-12 | p.Ala89Thr | rs2073451 | |
| IGLJ3 | p.Pro10Ala | rs2009433 | |
| MEI1 | p.Gly507Glu | rs267606261 | COSM36800 |
| NHS | p.Arg373Gln | rs267606412 | COSM1118631,COSM36761 |
| FGD1 | p.Arg636Trp | | COSM21850 |
| PJA1 | p.His586Tyr | rs267606501 | |
| OGT | p.Leu367Ser | | |
| DCAF12L1 | p.Ser281Phe | rs267606338 | COSM36778 |

## Disclaimer

This report is intended as a hypothesis generating framework and is thus intended for research use only and not for diagnostic or clinical purposes. Information provided in this report does not replace a physician's medical judgement and usage is entirely at your own risk. The providers of this resource shall in no event be liable for any direct, indirect, incidental, consequential, or exemplary damages.
