# Supplementary material for: ClinVAP: a reporting strategy from variants to therapeutic options
Source: Bioinformatics. 2019 Dec 12;36(7):2316–7. doi: 10.1093/bioinformatics/btz924 (PMC7141851; doi:10.1093/bioinformatics/btz924)
Supplement: btz924_Supplementary_Data [file btz924_supplementary_data.zip › btz924-suppl_data/SupplementaryMaterialC_Template.docx]

| **Patient Data** |
| --- |
| **Patient** {patient_lastname} {patient_firstname} |
| **Birthdate** {patient_dateofbirth} |
| **Diagnosis** {patient_diagnosis_short} |

| Mutation load {mutation_load} | Number of non-synonymous SNVs {mutation_ns_snv} |
| --- | --- |
| Number of oncogenes {mutation_affected_oncogenes} | |
| Number of tumor suppressor genes {mutation_affected_tumorsupressorgenes} | |
| Additional information {mutation_additional_information} | |

| **Somatic Mutations in Known Driver Genes** | | | | |
| --- | --- | --- | --- | --- |
| List of cancer driver genes along with the mutations observed in the patient. Confidence column shows the number of the driver gene sources that cataloged the corresponding gene as driver and Reference column gives the list of those sources. | | | | |
| **Gene** | **Mutation** | **Driver Type** | **Confidence^[[1]](#footnote-1)^** | **Reference** |
| {#mskdg}{Gene} | {Mutation} | {Type} | {Confidence} | {References}{/mskdg} |

| **Somatic Mutations with Known Pharmacogenetic Effect** | | | | | | | |
| --- | --- | --- | --- | --- | --- | --- | --- |
| List of drugs with the evidence of targeting the observed variant of the mutated gene regardless of the cancer type. The information is obtained from CIViC database. CIViC evidence levels are given in the Evidence column. | | | | | | | |
| **Gene** | **Mutation** | **Therapy** | **Effect** | **Disease** | | **Evidence^[[2]](#footnote-2)^** | **References** |
| {#mskpe}{Gene} | {Mutation} | {Therapy} | {Effect} | {Disease} | {Evidence} | | {/mskpe}{References} |

| **Somatic Mutations in Pharmaceutical Target proteins** | | | | | | |
| --- | --- | --- | --- | --- | --- | --- |
| **CIViC Summary of Drugs Targeting Affected Genes** | | | | | | |
| Therapies that have evidence of targeting the affected gene. The information is obtained from CIViC database. CIViC evidence levels are given in Evidence column. Results are filtered according to cancer type, if it is provided in metadata. | | | | | | |
| **Gene** | **Mutation** | **Therapy** | **Effect** | **Disease** | **Evidence^[[3]](#footnote-3)^** | **References** |
| {#ptp_ia}{Gene} | {Mutation} | {Therapy} | {Effect} | {Disease} | {Evidence} | {/ptp_ia}{References} |

| **Summary of Cancer Drugs Targeting Affected Genes** | | | | |
| --- | --- | --- | --- | --- |
| List of cancer drugs targeting the mutated gene. Information is obtained from DrugBank, Therapeutic Target Database, IUPHAR, and Santos et al. | | | | |
| **Gene** | **Status** | **Therapy** | **Confidence^[[4]](#footnote-4)^** | **References** |
| {#ptp_da}{Gene} | {Status} | {Therapy} | {Confidence} | {References}{/ptp_da} |

| **References** | |
| --- | --- |
| The publications of the reference IDs given in the tables above. | |
| {#ref}{rowid} | {citation}{/ref} |

| **Appendix** | | | |
| --- | --- | --- | --- |
| All the somatic variants of the patient with their dbSNP and COSMIC IDs. | | | |
| **Gene** | **Mutation** | **dbSNP** | **COSMIC** |
| {#appendix}{Gene} | {Mutation} | {dbSNP} | {COSMIC}{/appendix} |

| **Disclaimer** |
| --- |
| This report is intended as a hypothesis generating framework and is thus intended for research use only and not for diagnostic or clinical purposes. Information provided in this report does not replace a physician’s medical judgement and usage is entirely at your own risk. The providers of this resource shall in no event be liable for any direct, indirect, incidental, consequential, or exemplary damages. |

1. Confidence shows the number of driver gene sources that includes the gene. The sources are Vogelstein et al., Rubio-Perez et al., TSGene DB, COSMIC DB, UniProt. [↑](#footnote-ref-1)
2. [CIViC evidence levels are used](https://civicdb.org/help/evidence/evidence-levels). A = Validated association, B = Clinical evidence, C = Case study, D = Preclinical evidence, E = Inferential association [↑](#footnote-ref-2)
3. [CIViC evidence levels are used](https://civicdb.org/help/evidence/evidence-levels). A = Validated association, B = Clinical evidence, C = Case study, D = Preclinical evidence, E = Inferential association [↑](#footnote-ref-3)
4. Confidence shows the total number of the publications supporting the association. [↑](#footnote-ref-4)
